# Diversity and clinical correlations of SARS-CoV-2 variant during the introduction of the Delta variant in Guatemala

**DOI:** 10.1099/acmi.0.000939.v3

**Published:** 2026-03-03

**Authors:** Claudia Carranza, Lucia Ortiz, Maria Eugenia Castellanos, Ana Silvia Gonzalez-Reiche, Renata Mendizabal-Cabrera, Zain Khalil, Adriana van DeGuchte, Keith Farrugia, Mariana Herrera, Ernesto Mena, Celia Cordon-Rosales, Harm van Bakel, Daniel R. Perez

**Affiliations:** 1 Molecular Biology Laboratory, TecniScan de Guatemala S.A., Guatemala City, Guatemala; 2 Department of Population Health, Poultry Diagnostic and Research Center, College of Veterinary Medicine, University of Georgia, Athens, GA, USA; 3 Centro de Estudios en Salud, Universidad del Valle de Guatemala, Guatemala City, Guatemala; 4 College of Medicine and Dentistry, James Cook University, Townsville, Australia; 5 Department of Genetics and Genomic Sciences, Icahn School of Medicine at Mount Sinai, New York, NY, USA

**Keywords:** Delta variant, Guatemala, severe acute respiratory syndrome coronavirus 2 (SARS-CoV-2) surveillance

## Abstract

Severe acute respiratory syndrome coronavirus 2 (SARS-CoV-2) genomic surveillance is crucial for understanding viral evolution and guiding public health responses. However, many countries, particularly in Central America, have limited sequencing capacity, resulting in scarce and delayed data. This study addresses this gap by analysing 320 SARS-CoV-2 genomes sequenced from a major diagnostic centre in Guatemala City, Guatemala, between April and August 2021. Clade 21J (Delta) was predominant (46.2%), followed by 19B (29.4%) and 20J (Gamma, 6.6%). The most reported symptoms were cough, headache, malaise and myalgia/arthralgia. Among patients infected with the Delta variant, 39.9% reported being contacts from a confirmed case, less than reported by the patients infected with non-Delta variants (53.2%, *P*=0.017). The proportion of signs and symptoms was similar among these two groups, except for the history of fever, which was increased by ~twofold in the Delta group. This research contributes valuable genomic and epidemiological data to elucidate SARS-CoV-2 variant dynamics in Central America and emphasizes the importance of global genomic surveillance for pandemic preparedness and response.

## Data Summary

Genomic sequences were deposited in GenBank accession numbers OR075003–OR075050.

## Introduction

The emergence of severe acute respiratory syndrome coronavirus 2 (SARS-CoV-2) in December 2019 marked the onset of a global health crisis, prompting an unprecedented scientific response. Within months, the novel coronavirus rapidly spread worldwide, declared a pandemic by March 2020 [1,3]. The scientific community swiftly harnessed cutting-edge sequencing technologies, leading to the publication of the first complete SARS-CoV-2 genome in January 2020 [4]. This achievement, primarily driven by research efforts in high-income countries, laid the foundation for understanding the virus’s genetic makeup and its evolutionary trajectory.

However, as the virus spread across continents, it became evident that the genomic surveillance landscape was disproportionately skewed towards affluent nations [5]. The genomic data generated, while invaluable, primarily reflected the viral diversity circulating in resource-rich settings, leaving a significant blind spot in our understanding of SARS-CoV-2 evolution in low- and middle-income countries (LMICs). This disparity in data availability has far-reaching implications, hindering our ability to track the emergence of variants, assess their impact on disease severity and transmissibility and tailor public health interventions to specific regional contexts.

Latin America, bearing a substantial burden of COVID-19 cases and fatalities, exemplifies this data disparity [6]. The case fatality rate (CFR) for COVID-19 in Latin America ranged from 0.8% in Cuba to 6.2% in Peru by February 2022. Guatemala reported a CFR of 2.3% [7], illustrating regional disparities in the response to the pandemic, influenced by health system capacity, the progress of vaccination campaigns and the accuracy of reporting [8]. Despite concerted efforts by local researchers and public health agencies, the region’s contribution to global genomic surveillance remains disproportionately low. Studies conducted in Guatemala, a country significantly affected by the pandemic (2919.8 cases per 100,000 inhabitants in 2021) [9], have identified unique mutations in circulating SARS-CoV-2 strains, underscoring the importance of regional genomic data for understanding viral adaptation and transmission dynamics [10,11]. However, comprehensive genomic and epidemiological analyses in Guatemala, as in many other LMICs, are scarce, leaving critical questions unanswered.

While the National Health Laboratory in Guatemala has sequenced a substantial number of SARS-CoV-2 genomes, the inconsistent sampling rate over time and lack of public availability of this information raises concerns about the representativeness of this dataset for robust epidemiological inferences [12]. This study aims to bridge this gap by analysing genomic sequences from a randomly selected cohort of SARS-CoV-2 viruses collected in Guatemala between April and August 2021. By providing a detailed genomic and epidemiological characterization of these viral isolates, we seek to enhance our understanding of the molecular epidemiology and virus diversity of SARS-CoV-2 in Guatemala, contributing valuable insights to the global genomic surveillance landscape and informing public health strategies in the region.

## Methods

### Study site and design

This was a retrospective observational study, using nasopharyngeal swab samples collected from patients diagnosed with SARS-CoV-2 from TecniScan de Guatemala S.A., located in Guatemala City, Guatemala, during the surveillance period from 19 April 2021 to 31 August 2021. Each patient filled out a demographic, clinical and epidemiological survey designed by the Ministry of Health (see File S1, available in the online Supplementary Material). This study was approved by the Research Ethics Committee of the Center for Health Studies at Universidad del Valle de Guatemala (protocol number 251-08-2021).

### Sample selection for sequence data analysis

From 3,925 positive samples for SARS-CoV-2 from 15 April to 31 August 2021, we randomly selected a subset for sequence data analysis. We excluded duplicate samples with the same identifiers, as well as samples lacking identifiers. Also, we excluded samples with real-time PCR (RT-PCR) cycle threshold (Ct) values above 30, as the World Health Organization guidelines for SARS-CoV-2 genomic sequencing recommend that whole-genome sequencing be carried out in samples with RT-PCR Ct values up to 30 to achieve the highest number of samples with a full genome sequence [13]. Based on these exclusion criteria, we excluded a total of 234 samples, resulting in 3,691 samples available to be used for the sequence data analysis. After the previous step was completed, we randomly selected 442 samples (12%) using the R package *splitstackshape* [14]. The *splitstackshape* package allowed us to conduct stratified random sampling, in which the week of the sample diagnosis was the stratification (grouping) variable. That way, we were able to produce a list of randomly selected samples that represented 12% of the available samples per week. The random selection was carried out following the European Centre for Disease Prevention and Control guidelines, which recommend sequencing at least 10% of the positive test results [15]. We increased the value to 12% to account for samples that might have been lost in storage or handling. This sampling strategy was employed to track trends and estimate the distribution of variants with greater accuracy, including those with smaller proportions [16].

### Viral detection for SARS-CoV-2

Patients were diagnosed as SARS-CoV-2 positive by a RT-PCR isothermal assay for SARS-CoV-2 RNA detection, which was performed using the iAMP^®^ COVID-19 Detection Kit (Atila Biosystems) according to the manufacturer’s instructions. This kit allows the detection of SARS-CoV-2 RNA directly from swab samples without prior RNA extraction, detecting simultaneously the N and/or ORF1ab genes from the SARS-CoV-2 virus and an internal control using fluorescent probes labelled with FAM and HEX probes, respectively. RT-PCR reactions were conducted either in a Power gene 9600 thermocycler (Atila Biosystems, CA, USA) or MyGo thermocycler (Novacyt, UK) with the following thermal conditions: denaturation: 1 cycle at 61 °C for 30 s, followed by 50 cycles at 61 °C for 1 min. The supernatant of swabs was stabilized using RNAshield (Zymo Research, CA, USA) and stored at −20 °C for further analysis.

### SARS-CoV-2 amplification and sequencing

Virus RNA was extracted from 50 µl of supernatant using the MagMAX‐96 AI/ND Virus RNA Isolation Kit (Ambion, Austin, TX) according to the manufacturer’s instructions. All extracted RNA was stored at −70 °C until use. Viral RNA samples were sequenced by Illumina at the Icahn School of Medicine at Mount Sinai, using a targeted SARS-CoV-2 sequencing protocol with tiling primers that generates ~1.5 and ~2 kb amplicons with ~200 bp overlaps between each region, as previously described [17]. Sequencing protocol and genome assembly are described elsewhere [17,18].

### Phylogenetic and mutation prevalence analysis

**Fig. 1.**
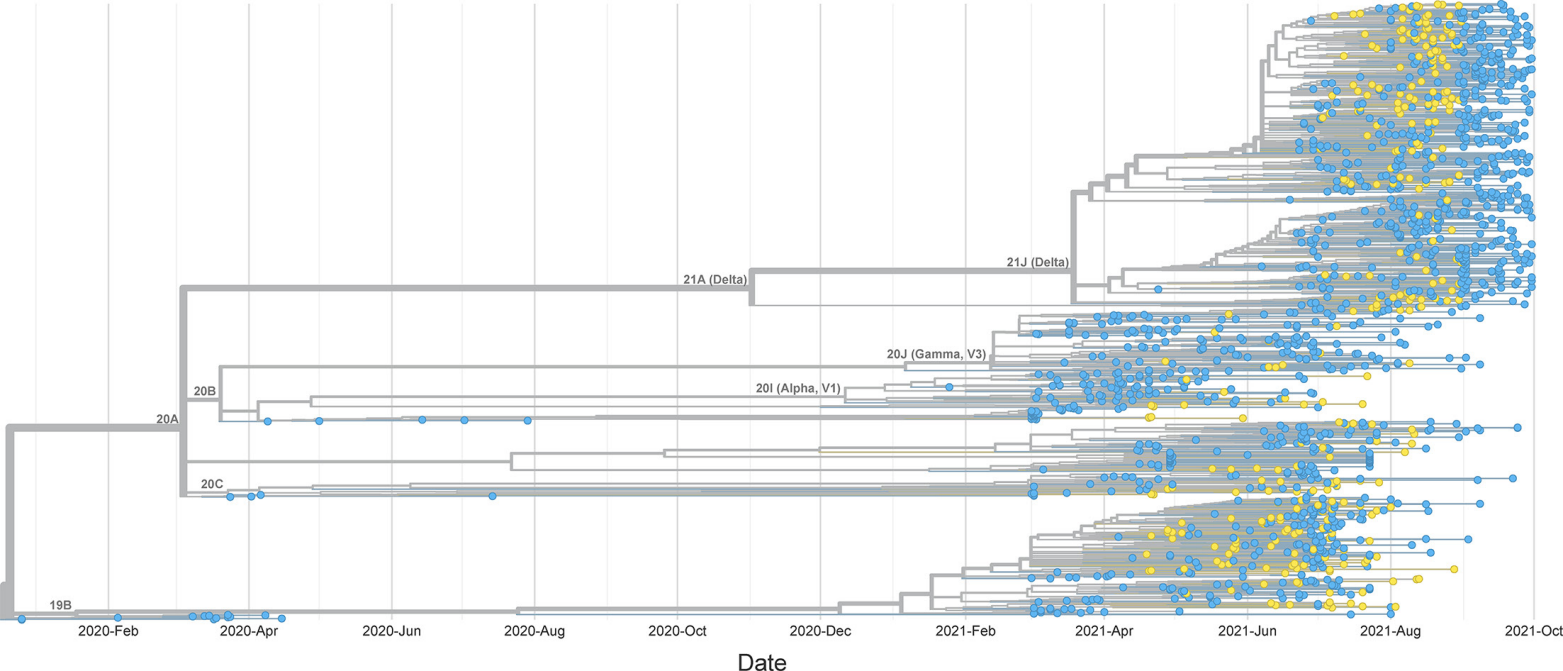
Time-scaled maximum likelihood phylogenetic tree of SARS-CoV-2 in Guatemala from April 2021 to August 2021 generated by Nexstrain. The tree shows 320 strains from Guatemala obtained for this study (yellow) combined with 1,024 strains from Central America (from the GISAID EPICoV database) between December 2019 and October 2021. The length of the branches represents the distance in time.

To generate a phylogenetic and divergence tree (Fig. 1), we downloaded all available 1,024 genome sequences of SARS-CoV-2 strains collected in Central America (downloaded in August 2022, dataset ID: EPI_SET_260226gc, https://doi.org/10.55876/gis8.260226gc) during April–August 2021 from the GISAID EpiCoV database [19] (https://www.gisaid.org). The sequences were analysed using NextStrain tools (https://nextstrain.org), including NextClade V1.6.0 [20]. SARS-CoV-2 lineage assignments were performed using the Phylogenetic Assignment of Named Global Outbreak LINeages nomenclature (Pangolin) COVID-19 Lineage Assigner [21,23] (https://gitlab.com/cgps/cog-uk/pangolin.cog-uk.io; https://cov-lineages.org). Mutations were identified using the GISAID CoVSurver (https://www.gisaid.org/epiflu-applications/covsurver-mutations-app/). The hCov-19/Wuhan/WIV04/2019 strain was used as a reference (Accession number NC-045512.2).

### Statistical analysis

**Fig. 2.**
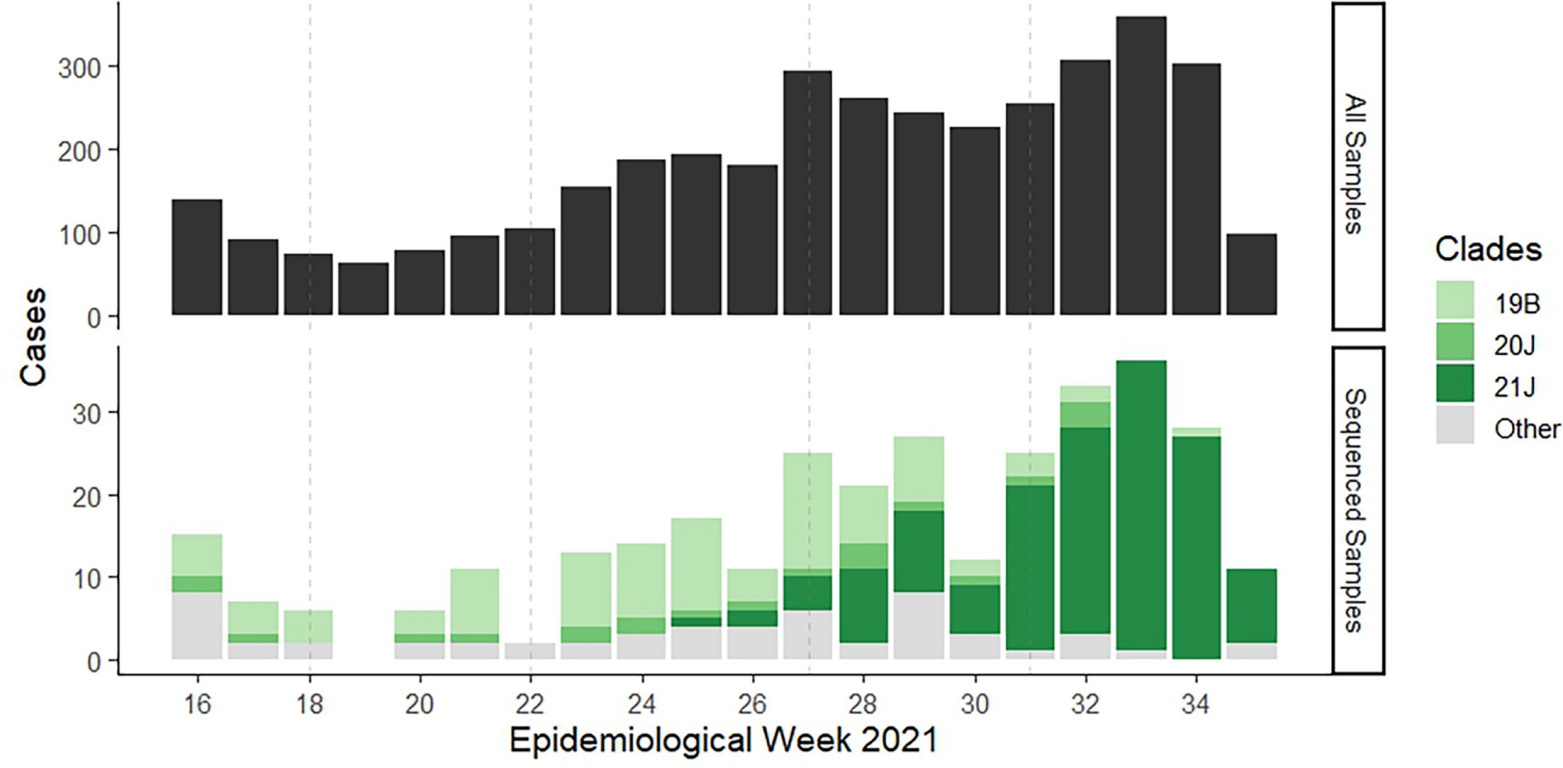
Distribution of SARS-CoV-2 samples and sequenced by variant from a major diagnostic centre in Guatemala during April–August 2021. Top plot: all positive SARS-CoV-2 samples available for sequence data analysis (*N*=3,691). Bottom plot: sequenced samples (*n*=320) showing clades. Category ‘Other’ includes 19A, 20A, 20B, 20C, 20G, 20I (Alpha, V1), 21C (Epsilon), 21F (Iota), 21G (Lambda), 21H (Mu), recombinant. SARS-CoV-2 cases are shown per epidemiological week.

**Fig. 3.**
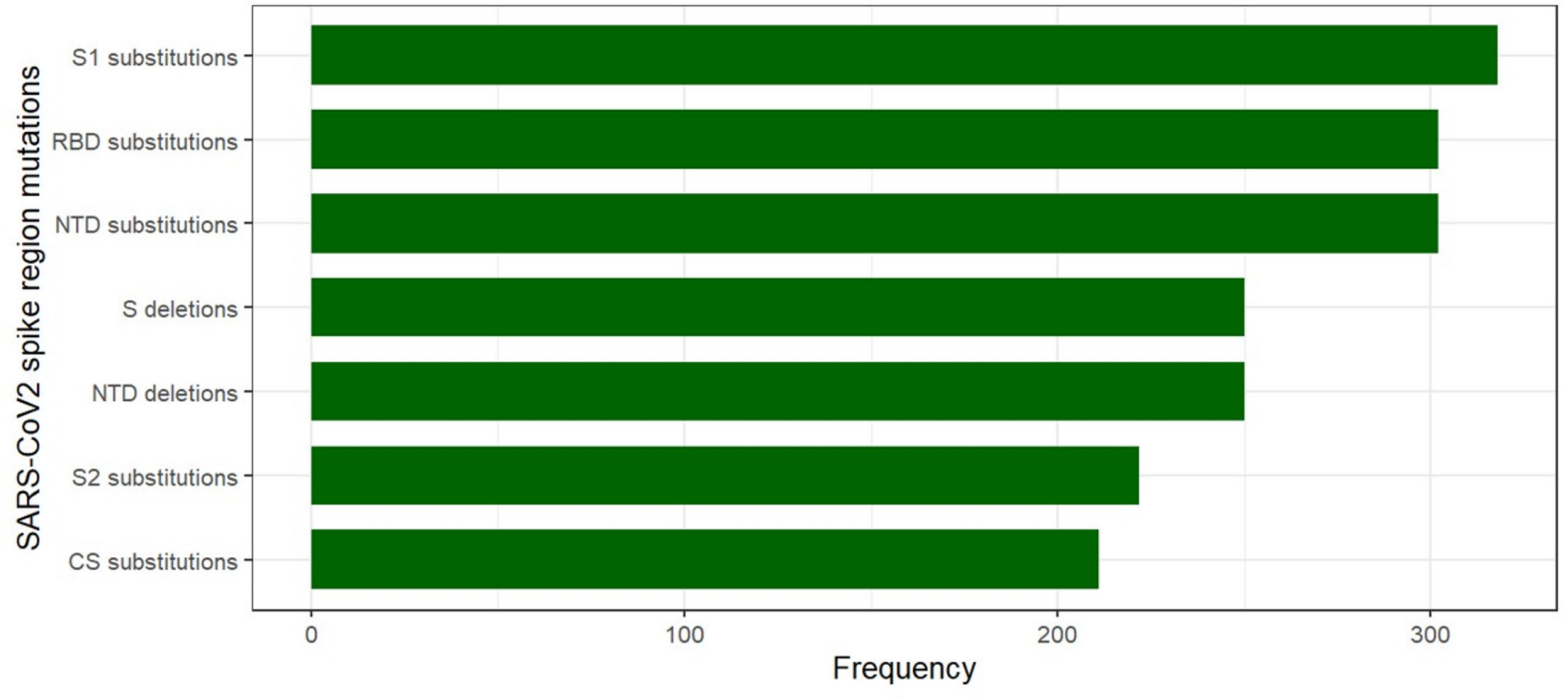
Absolute frequency of spike protein subregions mutations of SARS-CoV-2 in Guatemala.

Proportions and measures of central tendency were used to describe the demographic, clinical and epidemiological characteristics of the patients with fully sequenced genotype data. We calculated the total number of reported signs/symptoms by counting the presence/absence of the 14 related items listed in the survey. Descriptive statistics were used to summarize the distribution of the clades (overall and by month of diagnosis), as well as the frequency of the mutations in the spike region of SARS-CoV-2 in the samples. To assess the association between the Delta/non-Delta variants and categorical characteristics, the Chi-square test and Fisher’s exact test were used as appropriate. We used the Wilcoxon rank sum test for associations with continuous variables. All analyses were conducted using R version 4.2.2 and R Studio 2022.12.0.353. The *gtsummary* package [24] was used to create and report the descriptive results and summary tables. Figs 2 and 3 were created using the *ggplot2* package [25].

## Results

### Demographic and clinical profile of SARS-CoV-2 positive patients with successful genome sequencing in Guatemala

A total of 442 clinical samples from the 3,691 SARS-CoV-2 positive patients from 15 April to 30 August 2021 were randomly selected for sequence data analysis. However, 77 samples were not located, and 45 were not successfully sequenced. Therefore, 320 were sequenced (overall, 8.7% of positive samples selected were sequenced).

The median Ct value on these 320 samples was 13.8 cycles (IQR 11.8, 16.8). Patients with sequence data had a median age of 36 years (IQR 26, 47), and 51.4% were women (Table 1). Almost half of the patients reported being in contact with a confirmed case (47%), and 9.4% reported seeking medical attention. Only a small number of patients were healthcare workers (0.9%); most patients did not have diabetes (99.1%), and none reported hypertension. A majority of the patients were symptomatic (88.4%). The median number of reported symptoms among symptomatic patients was 3 (IQR 1, 5). The most common symptoms reported were cough (54.2%), headache (47.0%), malaise (43.1%) and myalgia or arthralgia (40.6%). Other symptoms less frequently reported were current fever (26.0%), rhinorrhoea (24.8%), odynophagia (21.0%), history of fever (20.7%) and loss of smell (19.1%).

**Table 1.** Demographic, clinical and epidemiological characteristics of 320 genotyped COVID-19 cases

Characteristic	*N*=320*
**Demographics and Epidemiology**	
Gender	
Female	161 (51.4%)
Male	152 (48.6%)
Age (yrs)	
Median (IQR)	36 (26, 47)
Contact from a confirmed case	150 (47.0%)
Medical attention asked	30 (9.4%)
Returned from abroad	5 (1.6%)
Diabetes	3 (0.9%)
Healthcare worker	3 (0.9%)
Hypertension	0 (0.0%)
Asymptomatic	37 (11.6%)
**Signs and symptoms**	
Number of reported symptoms	
Median (IQR)	3.00 (1.00, 5.00)
Cough	173 (54.2%)
Headache	150 (47.0%)
Malaise	137 (43.1%)
Myalgia or arthralgia	129 (40.6%)
Current fever	83 (26.0%)
History of fever	66 (20.7%)
Rhinorrhoea	79 (24.8%)
Odynophagia	67 (21.0%)
Loss of smell	61 (19.1%)
Loss of taste	17 (5.3%)
Vomiting or diarrhoea	27 (8.5%)
Dyspnoea	22 (6.9%)
Laryngeal stridor	8 (2.5%)
Subcostal retraction	4 (1.3%)

*n (%).

### Prevalence and temporal dynamics of SARS-CoV-2 lineages in Guatemala: dominance of Delta variant and absence of Omicron in mid-2021

The full-length SARS-CoV-2 virus genome was sequenced in 302 samples (genome completeness >99%), whereas 18 samples yielded incomplete genomes (genome completeness ranging from 3 to 93%). Incomplete genome sequences included those with miscalled bases at various genome positions due to sequencing errors. The mean genome size of isolated viruses was 29.6 kb (range 28.8–29.7 kb), with >90% covering the coding region. For this study, SARS-CoV-2 viruses were classified into major clades as defined by Nextstrain (https://clades.nextstrain.org). Clade 21J (Delta) was the most common variant (46.2%), followed by 19B (29.4%) and 20J (Gamma, 6.6%) (Fig. 1). The remaining 57 cases (17.8%) corresponded to at least 11 clades. The distribution of the three main clades varied according to the month of diagnosis (*P*<0.0001). During April, May and June 2021, the most frequent clade was 19B (Figs1  2). The Delta variant appeared for the first time in June 2021 (1 case) and in August became dominant (87% of all August cases) (Fig. 2). During the study period, no Omicron variants were detected.

### Comparative analysis of demographics and clinical manifestations in patients infected with Delta and non-Delta SARS-CoV-2 variants in Guatemala

In terms of age and sex, patients infected with the Delta variant were similar to those infected with non-Delta variants (Table 2). Among patients infected with the Delta variant, 39.9% reported contact with a confirmed case (as indicated in the survey completed by the patients), which was significantly lower than the proportion reported among patients infected with non-Delta variants (53.2%, *P*=0.017). The proportion of signs and symptoms was similar among these two groups, except for the history of fever which was increased by ~twofold in the Delta group (Table 2).

**Table 2.** Demographic and clinical characteristics by SARS-CoV-2 variant

Characteristic	Non-Delta, *N*=172*	Delta, *N*=148*	*P* value†
Gender			0.4
Female	91 (53.8%)	70 (48.6%)	
Male	78 (46.2%)	74 (51.4%)	
Age (years)			0.3
Median (IQR)	35 (26, 44)	38 (26, 51)	
Contact from a confirmed case	91 (53.2%)	59 (39.9%)	0.017
Medical attention asked	13 (7.6%)	17 (11.5%)	0.2
Returned from abroad	4 (2.3%)	1 (0.7%)	0.4
Diabetes	2 (1.2%)	1 (0.7%)	>0.9
Healthcare worker	2 (1.2%)	1 (0.7%)	>0.9
Asymptomatic	25 (14.6%)	12 (8.1%)	0.070
Number of reported symptoms			0.4
Median (IQR)	3.00 (1.00, 5.00)	3.00 (1.00, 5.00)	
Cough	87 (50.9%)	86 (58.1%)	0.2
Headache	80 (46.8%)	70 (47.3%)	>0.9
Malaise	74 (43.3%)	63 (42.9%)	>0.9
Myalgia or arthralgia	67 (39.4%)	62 (41.9%)	0.7
Current ever	46 (26.9%)	37 (25.0%)	0.7
History of fever	24 (14.0%)	42 (28.4%)	0.002
Rhinorrhoea	41 (24.0%)	38 (25.7%)	0.7
Odynophagia	36 (21.1%)	31 (20.9%)	>0.9
Loss of smell	36 (21.1%)	25 (16.9%)	0.3
Loss of taste	12 (7.1%)	5 (3.4%)	0.15
Vomiting or diarrhoea	13 (7.6%)	14 (9.5%)	0.6
Dyspnoea	15 (8.8%)	7 (4.8%)	0.2
Laryngeal stridor	4 (2.3%)	4 (2.7%)	>0.9
Subcostal retraction	3 (1.8%)	1 (0.7%)	0.6

*n (%).

†Pearson’s Chi-squared test; Wilcoxon rank sum test; Fisher’s exact test.

### Dominance of S1 subunit spike protein mutations and emergence of Delta variant in SARS-CoV-2 genomes from Guatemala

**Fig. 4.**
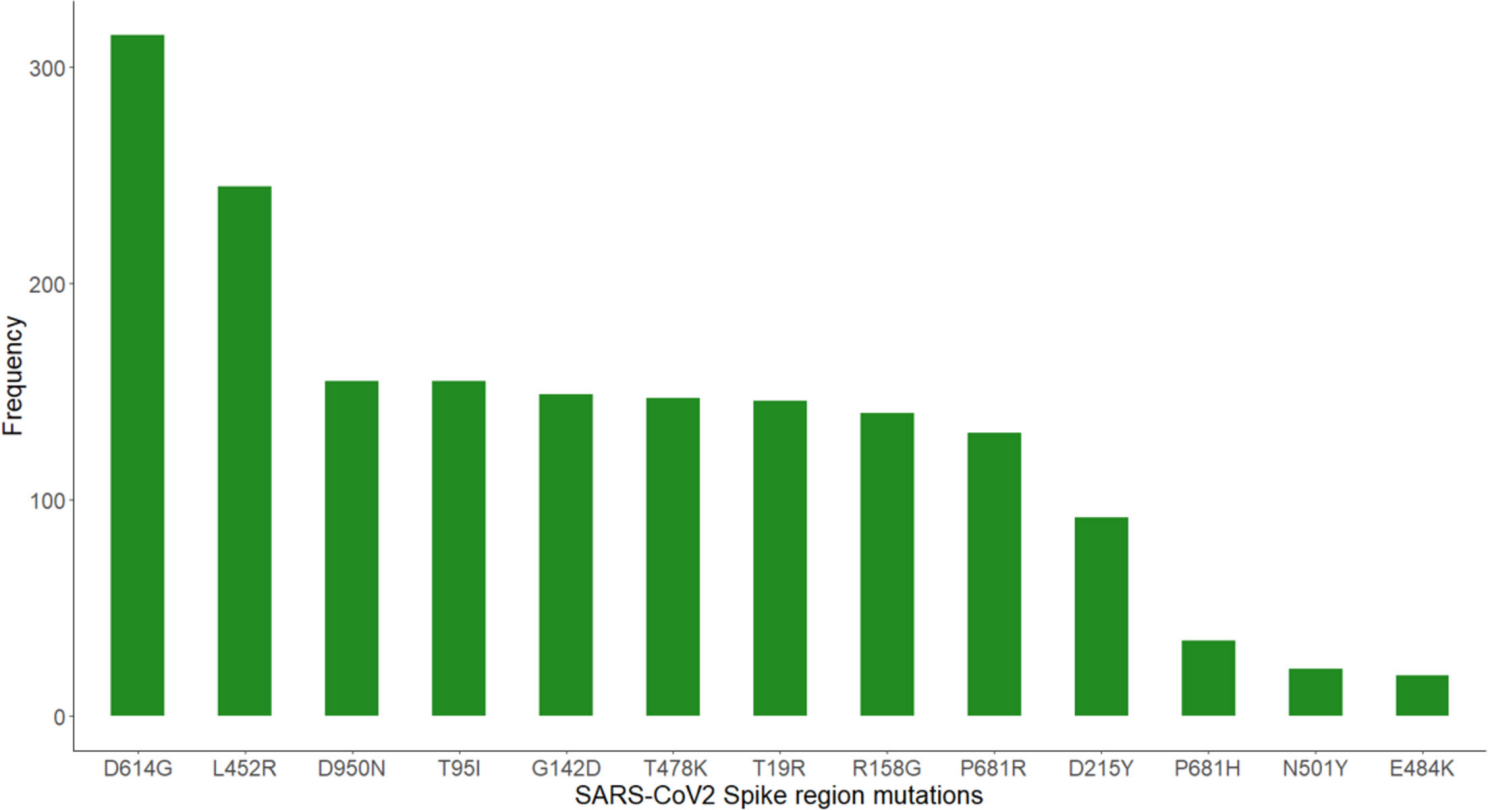
Absolute frequency of mutations found in this study during the evolution of SARS-CoV-2.

Amino acid substitutions were more common than deletions, with a mean of 26.75 substitutions per patient. In the spike protein, S1 subunit substitutions (*n*=318) were more frequent than S2 substitutions (*n*=222), whereas in the CS region, we found 211 substitutions. We found 302 substitutions in the C-terminal receptor binding domain (RBD) and 302 substitutions and 250 deletions in the N-terminal domain (NTD) (Fig. 3). We found a total of 2,340 missense mutations in the spike region. The most common mutations found in the S1 subunit were D614G (315/2,340), L452R (245/2,340), T95I (155/2,340), G142D (149/2,340), T478K (147/2,340), T19R (146/2,340), R158G (140/2,340), P681R (131/2,340) and D215Y (92/2,340) (Fig. 4). The most common mutation in the S2 subunit was D950N (155/2,340), and in region CS was P681R (131/2,340). For the Delta variant, we found the following mutations: L452R, D950N, G142D, P681R, D614G, T478K, T19R, R158G and T95I, whereas in the 19B clade, we found L452R, D215Y, D614G, A263S and W152R mutations. No new mutations were found in the present study. Most of the mutations found correspond to the variants found in this study, as well as to clade 19B.

## Discussion

This study sequenced 320 SARS-CoV-2 genomes from Guatemala between April and August 2021, revealing the dominance of the Delta variant (clade 21J) and a temporal shift in circulating clades. This aligns with global trends and national surveillance data indicating the emergence of a new clade [26,27]. The predominant clade was 21J, corresponding to the Delta variant, followed by 19B and 20J (Gamma variant). The Delta variant was first identified in India in October 2020, and it spread worldwide rapidly. This variant was highly transmissible compared to other variants, being more than 60% more transmissible than the Alpha variant [28]. In this study, the first cases of the Delta variant were detected in June 2021 and quickly became the predominant variant in July and August 2021, consistent with reports of molecular surveillance of the Ministry of Health of Guatemala that detected the Delta variant for the first time in the country in June 2021 with a maximum peak in August 2021 [27]. The variants that predominated in this study, before the Delta variant, were the Alpha variant and the Gamma variant, which were reported mainly in April, May and June 2021. Clade 19B was reported worldwide between January 2020 and August 2021 and was predominant in Guatemala in the April–June 2021 period. Clade 21J (Delta variant) was reported between March 2021 and March 2022, and according to data in this study, it began to circulate in Guatemala in June 2021, 3 months after the first reports in other regions. After these dates, the frequency of reporting of these clades decreased drastically [29,30]. Clades 19A and 19B were among the first SARS-CoV-2 clades described in humans, but they were immediately replaced worldwide by clade 20 in the early months of 2020. Clade 20A and its descendants contained the spike protein mutation D614G, which is associated with an increase in virus transmission [29]. In Quebec, Canada, cases infected by clade 19B virus were reported also between January and April 2021. This clade corresponds to lineage A.2.5.2 and was found in this period also in the USA, Panama and Costa Rica [31,32]. In France, at the beginning of the year 2021, a variant derived from clade 19B was reported. This variant is characterized by the presence of the following mutations in the spike protein: L18F, L452R, N501Y, A653V, H655Y, Q677H, D796Y and G1219V [15].

The Delta variant (21J clade) has important genomic features, including abundant mutations in the spike protein. The mutations present in the Delta variant increase cell-to-cell fusion, syncytia formation and cytopathic effects in cell cultures and greater pathogenic effects in the lungs of animal models [33]. The higher transmissibility of this variant has been associated with at least four mutations: D614G, L452R, P681R and T478K; all of them are also identified in sequences of this study. Two of these mutations (T484K and L452R) are located within the receptor-binding motif (RBM) of the spike protein and hence increase the affinity of RBD for angiotensin-converting enzyme 2, resulting in rapid viral attachment [34]. The mutation D614G, more frequently found in this study, significantly enhances the infectivity of the virus and causes a significant reduction in vaccine effectiveness [35,36]. Whereas L452R, the second most found mutation in this study, is located in the RBM region and is associated with improved virus transmission with a significantly increased severity of the disease and immune evasion [29,37,40].

Previous studies have shown that the most frequent symptoms of COVID-19 are fever, cough, fatigue and dyspnoea. Also, the US Center for Disease Control and Prevention recognized three main symptoms: fever, cough and shortness of breath. This list was expanded as the pandemic progressed to include chills, myalgia, headache, sore throat and loss of taste and smell [10,31, 34]. Clinical data analysis in this study revealed that the most frequent symptoms among the studied population were cough, headache and myalgia/arthralgia, similar to those reported in a study in California [41]. The only symptoms that differed among patients with Delta and non-Delta variants were the history of fever and being a contact of a confirmed case. Our findings are aligned with previous observations that Alpha and Delta variants had the same frequency of seven most common symptoms (fatigue, headache, anosmia/dysosmia, rhinorrhoea, sneezing, sore throat and cough) [42]. A subsequent study will evaluate the clinical and demographic data of the SARS-CoV-2 pandemic in Guatemala during the years 2020 and 2023. This study seeks to obtain information on whether symptoms and other variables have changed throughout the pandemic.

Scattered reports stemming from the SARS-CoV-2 pandemic are available from Central America, including Guatemala. These include reports of excess mortality during the COVID-19 pandemic in the country [43] and two other studies focused on agricultural workers and unvaccinated market workers [44,45]. Vaccine hesitancy in agricultural workers and higher risk of exposure to SARS-CoV-2 of market workers who interact with shoppers were the salient features of these studies. Additionally, the Ministry of Public Health and Social Assistance of Guatemala reported the number of confirmed, deceased and recovered COVID-19 cases over time, as well as data on partial genomic surveillance since May 2020 [10,27]. However, such data were not accompanied by systematic epidemiological analyses. The study presented here provides insights into the characteristics of the SARS-CoV-2 infection in the general population.

Despite this study’s valuable contributions, limitations include the short study period and the single-centre design, potentially affecting the generalizability of the findings. Nevertheless, this research underscores the critical need for continued molecular surveillance in Guatemala and the region to enhance our understanding of SARS-CoV-2 and future pathogens with epidemic potential. Our sampling strategy aimed to have ~12% of samples per week and was employed to track trends and estimate the distribution of variants with greater accuracy. However, we acknowledge that our selection criteria (only Ct values <30) and the fact that we could not sequence all the randomly selected samples may still introduce potential biases. In total, we were able to sequence 320 samples from a total of 3,691 (8.7%). This study contributes to the limited body of research on SARS-CoV-2 in Guatemala and Central America [43,45]. It offers valuable insights into the characteristics of the infection in the general population, complementing previous studies that focused on specific groups. Additionally, it emphasizes the importance of genomic surveillance in understanding viral evolution and guiding public health interventions [46].

## Supplementary material

10.1099/acmi.0.000939.v3 Supplementary File S1.

## Abbreviations

CFR: case fatality rate
Ct: cycle threshold
LMICs: low- and middle-income countries
RBD: C-terminal receptor binding domain
RBM: receptor-binding motif
RT-PCR: real-time PCR
SARS-CoV-2: severe acute respiratory syndrome coronavirus 2
