# Supplementary material for: Diversity and clinical correlations of SARS-CoV-2 variant during the introduction of the Delta variant in Guatemala
Source: Access Microbiol. 2026 Mar 3;8(3):000939.v3. doi: 10.1099/acmi.0.000939.v3 (PMC12956025; doi:10.1099/acmi.0.000939.v3)
Supplement: Supplementary File S1. [file acmi-8-00939-s001.pdf]

MINISTRY OF PUBLIC HEALTH AND SOCIAL ASSITANCE<br>NATIONAL CENTER FOR EPIDEMIOLOGY	EPIDEMIOLOGICAL FORM<br>RESPIRATORY VIRUS SURVEILLANCE – COVID 19	Version: 3<br>Update: march 2022

1. NOTIFICATION DETAILS

REPORTING UNIT	Date of notification	Day	Month	Year	Record No. (Health Unit Code)	Event COVID19	Year	No.
Health area				District				
Service or Hospital		Social security (IGSS)		Private Facility		Specify:		
Service where the case is received	Outpatient consultation		Emergency		Hospitalization		ICU		Residence		Other		Specify
Responsible for filling the form – Full name						Position		
Phone number		E-mail adress				Signature and seal		

2. PATIENT'S PERSONAL INFORMATION

**PATIENT'S NAME** First name				Second name				
First last name		Second last name		Sex	Masculine		Femenine	
Pregnant	Yes		No		Gestational age		Trimester	Post-partum	Yes		No	
Ocupation		Age	Years:	Months:	Date of birth	DAT	MONTH	YEAR
Patient's Personal Unique Identification Code (CUI)		Tutor's name		Tutor's phone number				
Community	Maya		Ladino/Mestizo		Garifuna		Xinca		Other: (specify)	
Education level	None		Elementary school		Middle school		High school diploma		Higher education		Unknown	

3. RESIDENTIAL ADRESS

**Address:**				Phone number	
Department		Municipality		Community/Village/Zone	

4. BACKGROUND

Suspected case of reinfection	Yes		No		Date of diagnostic		Place of diagnostic			
COVID-19 vaccine	Yes		No		Unknown		Number of doses received	One dose		Two doses		Three doses (booster shot)	
First dose vaccination date	Day	Month	Year	Type of vaccine received (1)		Source of vaccine information *(2)*				
Second dose vaccination date	Day	Month	Year	Type of vaccine received (1)		Source of vaccine information *(2)*				
Third dose vaccination date	Day	Month	Year	Type of vaccine received (1)		Source of vaccine information *(2)*				

5. CLINICAL DATA

RISK FACTORS

Diabetes mellitus	Yes		No		Unknown		Corticosteroid treatment	Yes		No		Unknown	
Chronic obstructive pulmonary disease (COPD)	Yes		No		Unknown		Chronic liver disease	Yes		No		Unknown	
Chronic renal failure	Yes		No		Unknown		Chronic cardiovascular disease (hypertension)	Yes		No		Unknown	
Cancer	Yes		No		Unknown		Neuromuscular dysfunction	Yes		No		Unknown	
Asthma	Yes		No		Unknown		Obesity	Yes		No		Unknown	
Inmunosupression	Yes		No		Unknown		Other:	Yes		No		Unknown	

**Onset date of symptoms**	Day	Month	Year	<<Symptoms at the time of sample collection>>>									
Fever ≥ 38°C	Yes		No		Unknown		Corticosteroid treatment	Yes		No		Unknown	
History of fever	Yes		No		Unknown		Chronic liver disease	Yes		No		Unknown	
General discomfort	Yes		No		Unknown		Chronic cardiovascular disease (hypertension)	Yes		No		Unknown	
Muscle or joint pain	Yes		No		Unknown		Neuromuscular dysfunction	Yes		No		Unknown	
Headache	Yes		No		Unknown		Obesity	Yes		No		Unknown	
Cough	Yes		No		Unknown		Subcostal retraction	Yes		No		Unknown	
Odynophagia	Yes		No		Unknown		Other:	Yes		No		Unknown	

6. COVID-19 INFORMATION

Reason for performing the COVID-19 test	Contact with confirmed case		Travel requirement	Seeking medical attention		Other		Specify:	
Did you participate in any social event with confirmed case(s) present in the last 14 days?	Yes		Specify:		No		Unknown		

7. SAMPLES COLLECTED

Sample was collected	Yes		No		Type of simple collected	Nasopharyngeal swab		Nasal swab		Sample collection date	Day	Month	Year
						Oropharyngeal swab		Combined swab					
Virus detected:													

8. EPIDEMIOLOGICAL SURVEILLANCE

Influenza-like Illness Surveillance – ILI (Symptomatic outpatient patients)		Severe Acute Respiratory Infections Surveillance – SARI (Symptomatic hospitalized patients)								
SARI/ILI in healthcare worker (symptomatic)							Yes		No	
Death related to SARI/ILI of unknown cause (symptomatic)							Yes		No	
Classification	Confirmed		Confirmed by epidemiological link		Suspected		Probable		Discarded	

9. PATIENT'S PROGRESS

The patient was hospitalized	Yes		No		Hospitalization date		Medical Record No.	

Admission to hospitalization	**Yes**		**No**		Admission to obseration	**Yes**		**No**	
Admission to ICU	**Yes**		**No**		Invasive mechanical ventilation	**Yes**		**No**	
					Non-nvasive mechanical ventilation	**Yes**		**No**	
Referred to another hospital	**Yes**		**No**		Which one?				

**Date of recovery/discharge/death**	Day	Month	Year	**Patient's condition at discharge**	**Alive**		**Deceased**	

(1) Type of vaccine:

* Pfizer Biontech	* Moderna	* Astra Zeneca/Oxford	* Sputnik	* Jhonson&Jhonson	* Others (specify)

(2) Source of Vaccine Information :

**1** Vaccination card reviewed in person	**2** Paper nominal registry	**3** National nominal registry (Electronic)	**4** Medical records	**5** Other PAI Records
**6** Vaccination card read by phone	**7** Verbal report without card	**8** No information		
